# Association of Peripheral Blood Neutrophil‐Lymphocyte Ratio With Motor and Cognitive Function in Prodromal Parkinson's Disease

**DOI:** 10.1002/brb3.71141

**Published:** 2025-12-17

**Authors:** Jin‐Sun Jun, Nyeonju Kang, Kyeongho Byun, Kiwon Park, Ryul Kim

**Affiliations:** ^1^ Department of Neurology Kangnam Sacred Heart Hospital Hallym University College of Medicine Seoul Republic of Korea; ^2^ Division of Sport Science Sport Science Institute & Health Promotion Center Incheon National University Incheon Republic of Korea; ^3^ Department of Biomedical and Robotics Engineering Incheon National University Incheon Republic of Korea; ^4^ Department of Neurology Seoul Metropolitan Government ‐ Seoul National University Boramae Medical Center Seoul National University College of Medicine Seoul Republic of Korea

**Keywords:** Parkinson's disease, neutrophil‐lymphocyte ratio, prodromal, REM sleep behavior disorder

## Abstract

**Introduction:**

Neutrophil and lymphocyte levels and their ratio, the neutrophil‐lymphocyte ratio (NLR), in the peripheral blood provide accessible indices of systemic inflammatory activity. In this study, we evaluated whether these blood markers are linked to motor symptoms and cognitive performance during the prodromal stage of Parkinson's disease (PD).

**Methods:**

We analyzed data from the prodromal cohort of the Parkinson's Progression Markers Initiative, which included 1,269 individuals with prodromal PD features (isolated REM sleep behavior disorder [RBD]: *n* = 116, isolated hyposmia: *n* = 570, combined: *n* = 583) and 284 healthy controls. Motor impairment was measured using the Movement Disorder Society Unified Parkinson's Disease Rating Scale Part 3, while cognition was assessed through a broad neuropsychological test battery.

**Results:**

Compared with controls, participants with prodromal PD had lower lymphocyte counts (*p* < 0.001) and higher NLR values (*p* = 0.008), whereas neutrophil counts did not differ between the groups (*p* = 0.749). No significant differences were observed across prodromal phenotypes for these markers. Correlation analysis demonstrated that NLR, but not neutrophil or lymphocyte counts alone, was associated with more severe motor impairment after false discovery rate correction (Q = 0.011). This relationship was evident in participants with both RBD and hyposmia and those with hyposmia only. However, the interaction between phenotype and NLR on motor severity was not significant. There were no significant associations of neutrophil or lymphocyte profiles with cognitive performance.

**Conclusion:**

Our findings indicate that higher NLR, largely reflecting reduced lymphocyte levels, is already present in the prodromal phase of PD and relates to the severity of motor symptoms. Prospective longitudinal studies are needed to clarify whether NLR has predictive value for progression to overt PD.

## Graphical abstract

Using data from the PPMI cohort, including 1,269 individuals with prodromal PD features (isolated RBD: n = 116, isolated hyposmia: n = 570, combined: n = 583) and 284 healthy controls, we found that in prodromal PD, NLR was elevated, primarily due to reduced lymphocyte counts, and this increase was associated with greater motor severity.

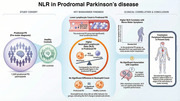

## 1 Introduction

Pathological alterations and neurodegeneration in Parkinson's disease (PD) usually develop long before the appearance of typical motor symptoms. During this prodromal period, patients may present with mild motor and cognitive dysfunction that does not meet the diagnostic criteria for parkinsonism or dementia, as well as a range of non‐motor features, such as rapid eye movement sleep behavior disorder (RBD), hyposmia, mood symptoms, and autonomic disturbances (Berg et al. 2021; Heinzel et al. 2019). Of these, isolated RBD (iRBD) is considered the most reliable prodromal marker identified thus far (Heinzel et al. 2019). Both the onset and the course of prodromal symptoms differ substantially across individuals (Hu et al. 2024), highlighting the importance of clarifying the factors that drive this heterogeneity in order to predict disease trajectories and mechanisms.

Neuroinflammatory processes are now recognized as important drivers of PD development and progression. Postmortem and imaging studies have revealed widespread microglial activation in the PD brain (Gerhard et al. 2006; McGeer et al. 1988; Ouchi et al. 2005), along with elevated inflammatory mediators across the central nervous system and the systemic circulation (Chen et al. 2018; Qin et al. 2016). Genetic studies also implicate immune and inflammatory pathways as significant contributors to PD susceptibility (Nalls et al. 2019; Pierce and Coetzee 2017). Importantly, neuroinflammatory signatures have also been observed in the prodromal stage. iRBD patients show microglial activation in the substantia nigra with concurrent nigrostriatal dopaminergic loss (Stokholm et al. 2017). In addition, proteomic profiling has revealed altered inflammatory proteins in iRBD (Hällqvist et al. 2024; Mondello et al. 2018), and longitudinal studies have linked peripheral cytokine patterns with the likelihood of phenoconversion (Kim et al. 2020; Zhang et al. 2020).

Neutrophil and lymphocyte levels and their ratio, the neutrophil‐lymphocyte ratio (NLR), in the peripheral blood serve as simple markers of systemic inflammation (Guthrie et al. 2013). Evidence increasingly points to their involvement in various neurodegenerative disorders, including PD. Meta‐analyses have reported higher NLR values in PD patients compared with controls (Hosseini et al. 2023; Muñoz‐Delgado et al. 2021), and Mendelian randomization analyses suggest that lower lymphocyte counts may causally increase PD risk (Jensen et al. 2021). Findings from the Parkinson's Progression Markers Initiative (PPMI) cohort demonstrated that higher neutrophil counts predicted more rapid motor deterioration and nigrostriatal dopaminergic loss (Kim et al. 2023). Despite these observations, the contribution of these inflammatory markers in the prodromal stage of PD remains poorly understood.

Accordingly, the present study aimed to evaluate neutrophil and lymphocyte counts as well as the NLR in individuals with prodromal PD compared with healthy controls, and examined how these markers relate to motor and cognitive outcomes in the prodromal stage.

## 2 Methods

### 2.1 Study Design and Participants

This analysis used data from the PPMI, a multicenter longitudinal project aimed at identifying biomarkers of PD development, with detailed protocols accessible at http://ppmi‐info.org (Pearson et al. 2025). Eligible participants included those with iRBD, individuals with isolated hyposmia, and healthy control subjects. iRBD participants were enrolled if the diagnosis was confirmed by polysomnography or by clinical evaluation from a sleep specialist. In cases without polysomnographic confirmation, enrollment required both hyposmia and an abnormal dopamine transporter (DAT) single‐photon emission computed tomography (SPECT) scan, defined either visually or quantitatively as below age‐expected values. Individuals with isolated hyposmia were eligible if they had a Parkinson's Associated Risk Syndrome (PARS) score ≤9 along with abnormal DAT‐SPECT findings (Jennings et al. 2017). The PARS score was calculated using an algorithm that integrated age, sex, and performance on the University of Pennsylvania Smell Identification Test. DAT‐SPECT scans were visually rated centrally by a nuclear medicine expert at the PPMI Imaging Core. To enlarge the control group, data from the early PD cohort were also included. Participants lacking blood cell count data, with a history of cancer or hematologic malignancy, or receiving immunosuppressive therapy were excluded. Moreover, carriers of known PD‐related genetic variants (including *LRRK2*, *GBA*, *SNCA*, *PRKN*, and *PINK1*) were not considered in this study, as these mutations may influence inflammatory profiles and exhibit patterns distinct from those observed in sporadic PD. All data used in this analysis were downloaded in July 2025.

Institutional review boards at all participating centers approved the study, and written informed consent was obtained from all participants. The study is registered at ClinicalTrials.gov (NCT04477785 and NCT01141023). The PPMI Data & Publication Committee approved publication of these analyses.

### 2.2 NLR Measurement

Complete blood counts were performed at Covance Laboratories using standardized PPMI protocols. The NLR was calculated by dividing the absolute neutrophil count (×10^3^/µL) by the absolute lymphocyte count (×10^3^/µL).

### 2.3 Clinical Variables

Motor severity was measured using the Movement Disorder Society–Unified Parkinson's Disease Rating Scale (MDS‐UPDRS) Part 3. Cognitive performance was evaluated with a standardized battery: Hopkins Verbal Learning Test (HVLT) for memory, Benton Judgment of Line Orientation (BJLO) for visuospatial function, F‐A‐S phonemic and semantic fluency (animals) for verbal fluency, Trail Making Test A (TMT‐A) and Symbol Digit Modalities Test (SDMT) for attention and processing speed, TMT‐B and Letter‐Number Sequencing (LNS) for executive and working memory, and the Boston Naming Test (BNT) for language. Raw scores were converted to standardized values using published norms.

### 2.4 Statistical Analysis

A normality test was performed with the Kolmogorov–Smirnov test. After confirming non‐normally distributed data for continuous variables and adequate expected cell counts for categorical variables, group differences were investigated with Mann–Whitney U tests for continuous variables and χ^2^ tests for categorical variables. Comparisons of neutrophil and lymphocyte counts and NLR values were conducted with nonparametric analysis of covariance (ANCOVA) adjusted for age, sex, body mass index (BMI), and regular use of non‐steroidal anti‐inflammatory drugs (NSAIDs) within 1 month prior to blood sampling. Within the prodromal PD cohort, associations between blood markers and clinical outcomes were assessed with Spearman rank correlations, in which raw values were automatically converted into ranks, with average ranks assigned for tied values according to the standard implementation. Although adjustments for multiple comparisons in these analyses were not primarily applied to minimize the risk of type II error given their exploratory nature, post‐hoc Benjamini–Hochberg false discovery rate (FDR) corrections across 11 tests were conducted as needed to evaluate the robustness of the findings. To assess whether the prodromal phenotype moderated the relationship between NLR and clinical variables, an interaction term (phenotype × NLR) was included in a linear regression model. Missing values were not imputed; instead, participants with missing data for a given variable were excluded from the corresponding analyses. Outliers were not removed, as the primary analyses employed non‐parametric methods that are relatively robust to the influence of extreme values. Additionally, no data transformations were applied. The two‐sided alpha level of 0.05 was used as the significance threshold for all statistical tests. For analyses involving multiple comparisons, FDR–adjusted *p*‐values were interpreted using the same threshold. Analyses were conducted using R, version 4.2.3 (R Foundation for Statistical Computing). Nonparametric ANCOVA was performed with the npANCOVA package, whereas all other analyses were conducted using base R functions.

## 3 Results

### 3.1 Study Population Characteristics

**FIGURE 1:**
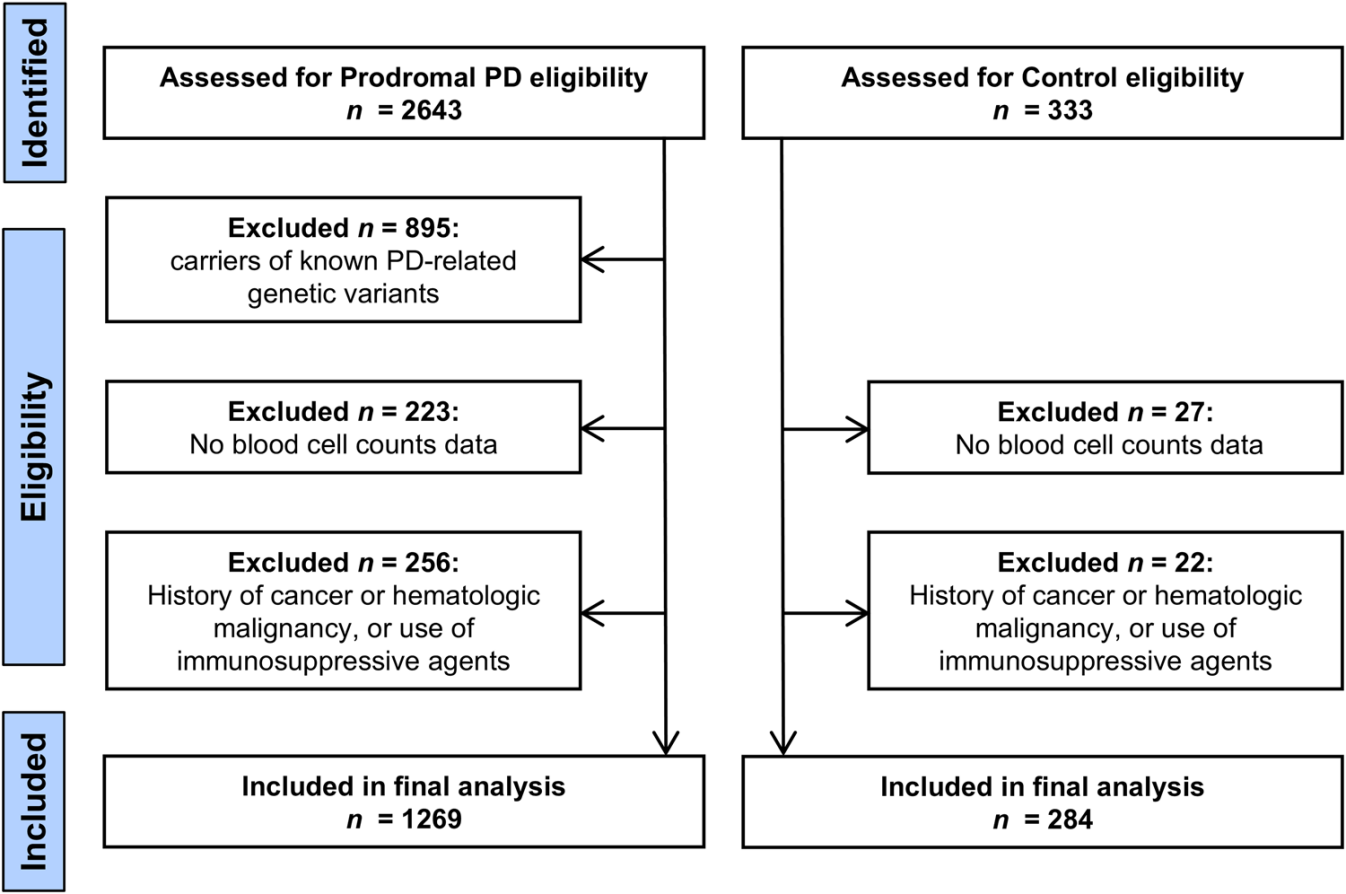
Flow diagram of participant selection Abbreviations: PD = Parkinson's disease.

After exclusions, 1269 participants with prodromal PD and 284 controls were included (Figure 1). Data were not missing for age, sex, total white blood cell count, neutrophil count, lymphocyte count, or the NLR. Data were missing for 10 patients for the MDS‐UPDRS Part 1, 3 patients for the MDS‐UPDRS Part 2, 23 patients for the MDS‐UPDRS Part 3, 6 patients for the HVLT‐delayed recall, 15 patients for the HVLT‐recognition discrimination, 15 patients for the BJLO, 15 patients for the phonemic fluency, 16 patients for the semantic fluency, 18 patients for the TMT‐A, 17 patients for the TMT‐B, 16 patients for the SDMT, 8 patients for the LNS, 28 patients for the BNT, and 20 patients for BMI. All missing data rates were below 3% across these variables. The median age of the prodromal PD group was 67.7 years (interquartile range, 63.9‐72.0), and 635 (50.0%) were men. Compared with controls, participants with prodromal PD were older, more often male, and had greater motor and cognitive impairment, particularly in memory and visuospatial domains, and higher BMI (Table 1). Demographic and clinical characteristics by prodromal phenotype are shown in Supplementary Table 1.

**TABLE 1:** Demographic, clinical, and laboratory characteristics of participants.

Variables	Prodromal PD	Controls	*p* value
Number of participants	1269	284	—
Age, years	67.7 (63.9–72.0)	63.3 (56.0–69.2)	<0.001^a^
Male sex, %	635 (50.0%)	163 (57.4%)	0.008
MDS‐UPDRS Part 1 score	6.0 (3.0–10.0)	2.0 (1.0–5.0)	<0.001^a^
MDS‐UPDRS Part 2 score	1.0 (0.0–3.0)	0.0 (0.0–0.0)	<0.001^a^
MDS‐UPDRS Part 3 score	3.0 (1.0–7.0)	0.0 (0.0–2.0)	<0.001^a^
HVLT‐delayed recall score	48.0 (37.0–56.0)	53.0 (44.0–58.8)	<0.001^a^
HVLT‐recognition discrimination score	52.0 (42.0–59.0)	51.0 (44.0–58.0)	0.738^a^
BJLO score	12.3 (10.6–14.2)	12.8 (11.1–14.8)	0.008^a^
PFT score	50.0 (43.0–57.0)	50.0 (46.0–61.0)	0.080^a^
SFT score	53.0 (46.0–58.5)	52.0 (47.0–58.0)	0.890^a^
TMT‐A score	63.6 (31.9–84.6)	68.1 (42.9–85.0)	0.129^a^
TMT‐B score	55.6 (12.1–81.8)	62.5 (25.1–86.0)	0.078^a^
SDMT score	51.3 (45.0–57.5)	51.0 (44.4–58.3)	0.497^a^
LNS score	12.0 (10.0–14.0)	12.0 (10.0–13.0)	0.350^a^
BNT score	12.0 (10.0–14.0)	12.0 (9.0–14.0)	0.602^a^
Body mass index, kg/m^2^	27.1 (24.3 30.5)	26.2 (23.6 29.6)	0.003^a^
NSAID use^*^, %	255 (20.1%)	67 (23.6%)	0.129
Total WBC count (×10^3^/µL)	5.61 (4.81–6.75)	5.81 (4.85–6.90)	0.152^b^
Neutrophil count (×10^3^/µL)	3.45 (2.84–4.35)	3.56 (2.83–4.31)	0.749^b^
Lymphocyte count (×10^3^/µL)	1.53 (1.25–1.87)	1.64 (1.35–2.05)	<0.001^b^
Neutrophil‐lymphocyte ratio	2.23 (1.73–2.96)	2.13 (1.64–2.71)	0.008^b^

The data are expressed as No. (%) or median value (interquartile range).

* Regular use within 1 month prior to blood sampling.

^a^ *P* values were obtained using the Mann–Whitney U test.

^b^ *P* values were obtained using nonparametric analysis of covariance.

**Abbreviations:** BJLO = Benton Judgment of Line Orientation; BNT = Boston Naming Test; HVLT = Hopkins Verbal Learning Test; LNS = Letter‐Number Sequencing; MDS‐UPDRS = Movement Disorders Society Unified Parkinson's Disease Rating Scale; PD = Parkinson's disease; PFT = Phonemic Fluency Test; SDMT = Symbol‐Digit Modalities Test; SFT = Semantic Fluency Test; TMT = Trail Making Test, WBC = white blood cell.

### 3.2 Blood Neutrophil and Lymphocyte Counts and the NLR in Prodromal PD

**FIGURE 2:**
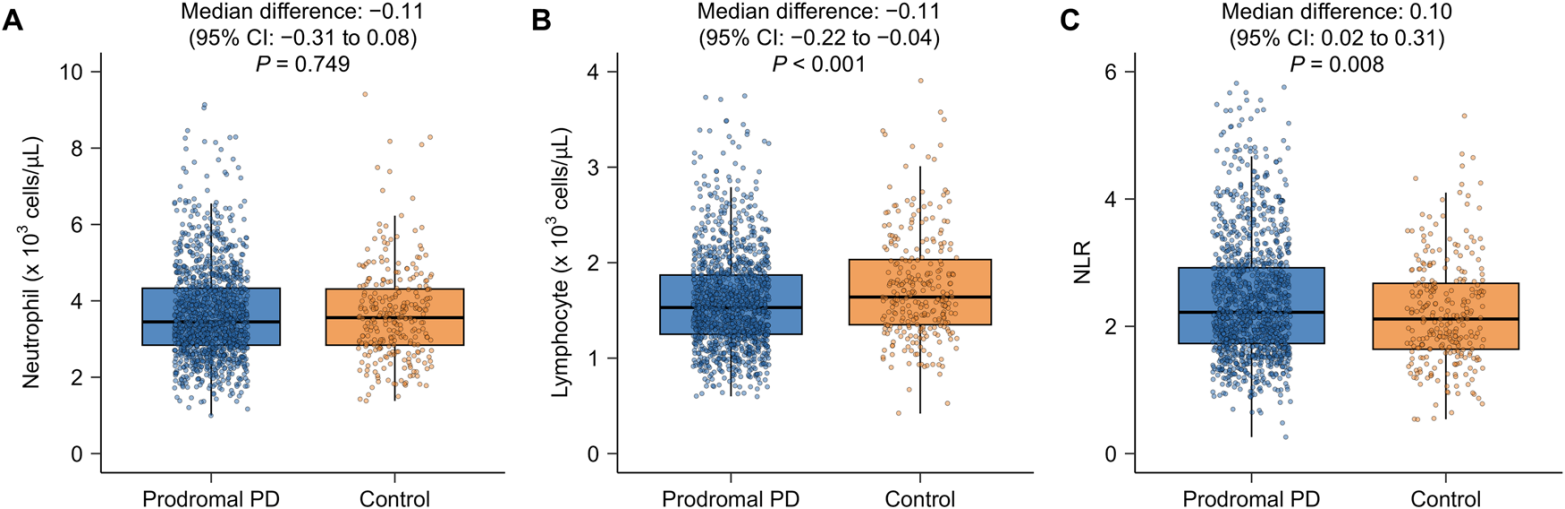
Comparisons of blood neutrophil and lymphocyte counts and the NLR between patients with prodromal PD and controls. For (A) neutrophil count, (B) lymphocyte count, and (C) NLR, the box plot shows the interquartile range, with the horizontal bar representing the median. Whiskers indicate the range from minimum to maximum values, while outliers >1.5 times the interquartile range are excluded. Abbreviations: CI = confidence interval; NLR = neutrophil‐to‐lymphocyte ratio; PD = Parkinson's disease.

**FIGURE 3:**
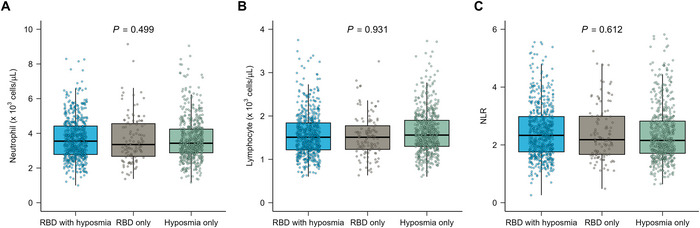
Comparisons of blood neutrophil and lymphocyte counts and the NLR according to distinct prodromal phenotypes. For (A) neutrophil count, (B) lymphocyte count, and (C) NLR, the box plot shows the interquartile range, with the horizontal bar representing the median. Whiskers indicate the range from minimum to maximum values, while outliers >1.5 times the interquartile range are excluded. Abbreviations: NLR = neutrophil‐to‐lymphocyte ratio; RBD = REM sleep behavior disorder.

Neutrophil and lymphocyte counts and NLR values are shown in Figure 2. In prodromal PD patients, age correlated positively with neutrophil counts (Spearman r = 0.061, 95% confidence interval [CI] = 0.005 to 0.114; *p* = 0.029) and NLR (Spearman r = 0.122, 95% CI = 0.066 to 0.175; *p* < 0.001) and negatively with lymphocyte counts (Spearman r = −0.099, 95% CI = −0.155 to −0.044; *p* < 0.001). Male sex was also associated with higher neutrophil counts (*p* = 0.041), lower lymphocyte counts (*p* < 0.001), and consequently higher NLR values (*p* < 0.001). After adjustment for age, sex, BMI, and regular use of NSAIDs, prodromal PD participants had lower lymphocyte counts (median difference, −0.11; 95% CI, −0.22 to −0.04; *p* < 0.001) and higher NLR values (median difference, 0.10; 95% CI, 0.02 to 0.31; *p* = 0.008) than controls, whereas neutrophil counts did not differ significantly (median difference, −0.11; 95% CI, −0.31 to 0.08; *p* = 0.749). No significant differences in neutrophil counts (*p* = 0.499), lymphocyte counts (*p* = 0.931), or NLR (*p* = 0.612) were found across prodromal subgroups (Figure 3).

### 3.3 Association of Blood Neutrophil and Lymphocyte Counts and the NLR With Clinical Variables in Prodromal PD

Bivariate correlation analyses are summarized in Table 2. Neutrophil counts were associated with lower SDMT performance (Spearman r = −0.080, 95% CI = −0.135 to −0.021; *p* = 0.025), lymphocyte counts with lower MDS‐UPDRS Part 3 scores (Spearman r = −0.069, 95% CI = −0.128 to −0.019; *p* = 0.016), and NLR with higher MDS‐UPDRS Part 3 scores (Spearman r = 0.094, 95% CI = 0.033 to 0.150; *p* = 0.001) and lower performance on HVLT‐delayed recall (Spearman r = −0.058, 95% CI = −0.115 to 0.000; *p* = 0.043), HVLT‐recognition discrimination (Spearman r = −0.069, 95% CI = −0.125 to −0.014; *p* = 0.013), and SDMT (Spearman r = −0.061, 95% CI = −0.120 to −0.004; *p* = 0.030). However, only the association between NLR and MDS‐UPDRS Part 3 remained statistically significant after FDR correction (Q = 0.011). Stratified analyses showed this association in the RBD with hyposmia and hyposmia only groups, but not in the RBD only group (Supplementary Table 2). The interaction between phenotype and NLR on MDS‐UPDRS Part 3 was not statistically significant (*p* = 0.617).

**TABLE 2:** Correlates of blood neutrophil and lymphocyte counts and NLR with outcome measures in patients with prodromal PD.

	Neutrophil		Lymphocyte		NLR	
Variables	Spearman r (95% CI)	*p* value	Spearman r (95% CI)	*p* value	Spearman r (95% CI)	*p* value
MDS‐UPDRS Part 3 score	0.021 (−0.035 to 0.073)	0.470	−0.069 (−0.128 to −0.019)	**0.016**	0.094 (0.033 to 0.150)	**0.001**
HVLT‐delayed recall score	−0.034 (−0.089 to 0.018)	0.244	0.023 (−0.031 to 0.077)	0.425	−0.058 (−0.115 to 0.000)	**0.043**
HVLT‐recognition discrimination score	−0.055 (−0.113 to 0.002)	0.055	0.054 (−0.004 to 0.112)	0.059	−0.069 (−0.125 to −0.014)	**0.013**
BJLO score	−0.048 (−0.110 to 0.002)	0.093	−0.049 (−0.108 to 0.004)	0.086	0.002 (−0.052 to 0.057)	0.941
PFT score	0.010 (−0.052 to 0.069)	0.729	−0.020 (−0.074 to 0.038)	0.492	0.013 (−0.049 to 0.068)	0.643
SFT score	−0.011 (−0.069 to 0.045)	0.696	0.045 (−0.012 to 0.100)	0.120	−0.049 (−0.104 to 0.009)	0.089
TMT‐A score	−0.030 (−0.084 to 0.032)	0.300	0.014 (−0.041 to 0.068)	0.625	−0.048 (−0.102 to 0.010)	0.097
TMT‐B score	−0.047 (−0.107 to 0.013)	0.106	−0.001 (−0.059 to 0.058)	0.982	−0.044 (−0.100 to 0.015)	0.123
SDMT score	−0.080 (−0.135 to −0.021)	**0.005**	0.012 (−0.046 to 0.063)	0.680	−0.061 (−0.120 to −0.004)	**0.030**
LNS score	−0.023 (−0.087 to 0.033)	0.419	−0.053 (−0.109 to 0.000)	0.067	0.020 (−0.041 to 0.078)	0.487
BNT score	−0.031 (−0.088 to 0.028)	0.279	−0.040 (−0.094 to 0.020)	0.168	0.004 (−0.056 to 0.057)	0.898

Bold text indicates a statistically significant difference.

**Abbreviations:** BJLO = Benton Judgment of Line Orientation; BNT = Boston Naming Test; CI = confidence interval; HVLT = Hopkins Verbal Learning Test; LNS = Letter‐Number Sequencing; MDS‐UPDRS = Movement Disorders Society Unified Parkinson's Disease Rating Scale; NLR = neutrophil‐lymphocyte ratio; PD = Parkinson's disease; PFT = Phonemic Fluency Test; SDMT = Symbol‐Digit Modalities Test; SFT = Semantic Fluency Test; TMT = Trail Making Test.

## 4 Discussion

In this study, we found that the NLR was significantly elevated in patients with prodromal PD compared with controls, consistent with findings from meta‐analyses in clinically manifest PD (Hosseini et al. 2023; Muñoz‐Delgado et al. 2021). Although this peripheral marker does not fully capture the complexity of underlying immune mechanisms, our findings provide potential evidence of neuroinflammation preceding overt motor signs and suggest that an exaggerated systemic immune response may contribute to disease pathogenesis. Nonetheless, increased NLR has also been observed in a range of neurodegenerative conditions, such as Alzheimer's disease (Sayed et al. 2020), Huntington's disease (Xia et al. 2024), multiple system atrophy (Zhang et al. 2022), and progressive supranuclear palsy (Inci et al. 2020), and the reported NLR levels across such disorders appear broadly similar in magnitude. These findings suggest that elevated NLR reflects a nonspecific inflammatory response rather than a disease‐specific signature. Furthermore, given the substantial overlap between prodromal PD and controls, NLR alone is unlikely to serve as a reliable diagnostic marker.

The increase in NLR was driven primarily by a reduction in lymphocyte counts rather than by elevated neutrophil counts. Similarly, lymphocyte count reduction has been reproducibly reported in previous studies of PD, whereas neutrophil count changes have shown conflicting results (Grillo et al. 2023; Li et al. 2024; Muñoz‐Delgado et al. 2021; Muñoz‐Delgado et al. 2023b; Kim et al. 2023; Zhang et al. 2024b). Accordingly, our findings suggest that lymphocyte reduction, rather than neutrophil elevation, may serve as a more sensitive peripheral marker of neuroinflammation during the prodromal stage of PD. The decline in circulating lymphocytes was mainly attributable to declines in CD4+ and CD8+ T cells, along with imbalances in subsets and functional dysregulation, notably favoring pro‐inflammatory phenotypes such as T helper (Th) 1 and Th17 cells (Jiang et al. 2017; Omata et al. 2025; Tan et al. 2020). In contrast, patients with PD exhibited elevated lymphocyte levels in the substantia nigra pars compacta (Brochard et al. 2009). We could not fully understand the underlying mechanisms, but previous studies indicate that microglia‐derived tumor necrosis factor (TNF)‐α facilitates lymphocyte migration from the peripheral circulation into the central nervous system via a compromised blood–brain barrier (Sayed et al. 2020). This migration is mediated by TNF‐α–induced chemoattraction and adhesion (Sayed et al. 2020). Interestingly, we observed a sex difference in NLR, with higher values in male PD patients, which is consistent with prior observations (Bovenzi et al. 2025). This pattern suggests that immune activation in PD may be more pronounced in men, potentially reflecting sex hormones‐related biological influence (Bovenzi et al. 2025).

Of note, among the blood biomarkers, only a higher NLR was associated with greater motor severity in patients with prodromal PD after adjustment for multiple testing, suggesting that motor severity in the prodromal stage of PD may be driven by complex immune alterations involving both innate and adaptive immunity rather than by isolated changes in a single cell type. In line with our findings, NLR has also been repeatedly associated with motor severity in clinically manifest PD (Grillo et al. 2023; Li et al. 2024; Yi et al. 2024). Although the mechanisms underlying the association between NLR and motor function during the prodromal stage remain to be fully elucidated, several plausible explanations have been proposed. An elevated NLR may serve as an indirect marker of central neuroinflammation, which is considered to accelerate motor progression in PD by promoting nigrostriatal dopaminergic degeneration (Tansey et al. 2022). Supporting this notion, previous studies have demonstrated an association between higher NLR and lower dopamine transporter binding in the striatum (Muñoz‐Delgado et al. 2023a; Zhang et al. 2024a). Alternatively, elevated NLR has been linked to greater white matter hyperintensity (Nam et al. 2017). In addition, evidence suggests a relationship between higher NLR and increased amyloid and tau deposition (Grillo et al. 2023; Jacobs et al. 2024; Mehta et al. 2023). Given that both white matter changes (Yang et al. 2023) and Alzheimer's pathologies (Chu et al. 2024; Kim et al. 2019; Park et al. 2024) are recognized contributors to motor progression in PD, such disease‐nonspecific mechanisms may exacerbate or accelerate motor progression during the prodromal stage.

The association between NLR and motor function was only observed in the RBD with hyposmia and hyposmia only groups. However, we found no clear evidence that the NLR–motor function relationship differed significantly across phenotypes in this cohort. The limited number of participants in the RBD only group may have reduced the sensitivity to detect phenotype‐specific effects and may account for the absence of a significant association in this subgroup. Additionally, not all participants in the hyposmia only group underwent polysomnography, raising the possibility of undetected RBD. Substantial overlap in prodromal risk features across these groups may have contributed to the lack of phenotype‐specific differences. Ideally, distinctions such as a “hyposmia‐first” versus “RBD‐first” trajectory would help clarify differential patterns, but such temporal information was not available in this cohort.

Since the course and features of early cognitive dysfunction in PD are highly variable (Aarsland et al. 2021), identifying biological markers that reflect this heterogeneity may offer valuable insights into the pathological processes underlying cognitive impairment. We therefore examined whether the NLR and its components were associated with cognition during the prodromal stage of PD. Our results showed that processing speed and attention showed trends toward associations with both neutrophil counts and the NLR, while memory performance showed a trend with the NLR alone; however, none remained significant after correction for multiple testing. Moreover, no significant associations were observed between lymphocyte counts and cognitive measures. This lack of association may reflect the overall mild severity of cognitive function in this cohort. Although several cognitive domains were significantly more impaired than in controls, the magnitude of these differences was modest. In particular, cognitive performance was largely within the normal range. This restricted variability likely resulted in a ceiling effect, reducing the sensitivity to detect meaningful correlations. To better delineate the impact of circulating neutrophils and lymphocytes in the prodromal stage of PD, further longitudinal studies including patients with a broader range of cognitive severity are warranted.

Major strengths of this study include strict inclusion and exclusion criteria, standardized data collection, and a large, well‐characterized cohort. To the best of our knowledge, this is the first investigation into the clinical implications of peripheral blood neutrophils and lymphocytes in individuals with prodromal PD. However, several limitations should be noted. First, our analysis was based on a cross‐sectional design with only one time‐point of data collection. Accordingly, causality between neutrophils or lymphocytes and clinical variables could not be determined. Patients in the PPMI cohort have been followed longitudinally to assess changes in clinical symptoms, and further analyses using these data may clarify the current findings. Second, we excluded patients with cancer or hematologic malignancy that could influence peripheral inflammatory marker levels, but the potential impact of unrecognized or residual confounding at blood draw, such as recent infection or other nonmalignant conditions, cannot be ruled out. Furthermore, lifestyle factors such as smoking status, alcohol consumption, and physical activity are potential confounders in the association between NLR and prodromal PD; however, we were unable to adjust for these variables because of insufficient or unavailable data. Third, further cohort studies including more ethnically and clinically diverse patient populations are needed to confirm the generalizability of our findings. Lastly, differences in age and sex distribution between the prodromal PD and control groups may have influenced our results, although all analyses were adjusted for these potential confounders.

In conclusion, we observed an elevation in NLR, primarily driven by reduced lymphocyte counts, in patients with prodromal PD, which supports the concept that peripheral inflammation is present in the early stage of the disease. Notably, a higher NLR was associated with greater motor severity during the prodromal stage. These findings highlight the need for longitudinal research to clarify whether NLR has predictive utility for the transition from prodromal to clinically established PD.

## Author Contributions

**Jin‐Sun Jun**: conceptualization, methodology, investigation, writing – original draft, formal analysis, supervision, project administration. **Nyeonju Kang**: methodology, writing – review and editing. **Kiwon Park**: methodology, writing – review and editing. **Kyeongho Byun**: methodology, writing – review and editing. **Ryul Kim**: conceptualization, methodology, writing – review and editing, supervision, project administration.

## Funding

This work was supported by the National Research Foundation (NRF) grant funded by the Korea government (MSIT) (Nos. 2021R1C1C1011822, RS‐2021‐NR061646, and RS‐2023‐00208906).

## Ethics Statement

Approval granted by the PPMI Data Access Committee by submitting a brief application detailing planned analyses, qualified researcher supervision and compliance with the Data Use Agreement (process: https://www.ppmi‐info.org/access‐data‐specimens/download‐data). No reference number/ID provided. Participants gave informed consent to participate in the study before taking part.

## Supporting information




**Supplementary Material**: brb371141‐Sup‐0001‐TableS1

**Supplementary Material**: brb371141‐Sup‐0002‐TableS2

## Data Availability

All data reported in this article are available in the PPMI repository (www.ppmi‐info.org).
