# Supplementary material for: Association of Peripheral Blood Neutrophil‐Lymphocyte Ratio With Motor and Cognitive Function in Prodromal Parkinson's Disease
Source: Brain Behav. 2025 Dec 17;15(12):e71141. doi: 10.1002/brb3.71141 (PMC12712348; doi:10.1002/brb3.71141)
Supplement: Supplementary file 1 — Supplementary Material: brb371141‐Sup‐0001‐TableS1 [file BRB3-15-e71141-s001.docx]

**Supplementary Table 1. Demographic, clinical, and laboratory characteristics according to distinct prodromal phenotypes**

| **Variables** | **RBD with hyposmia** | **RBD only** | **Hyposmia only** |
| --- | --- | --- | --- |
| Number of participants | 583 | 116 | 570 |
| Age, years | 68.6 (64.6–72.6) | 68.2 (62.2–72.4) | 66.9 (63.4–70.5) |
| Male sex, % | 377 (64.7%) | 94 (81.0%) | 164 (28.8%) |
| MDS-UPDRS Part 1 score | 7.0 (4.0–11.0) | 7.0 (4.0–10.0) | 5.0 (2.0–8.0) |
| MDS-UPDRS Part 2 score | 1.0 (0.0–4.0) | 1.0 (0.0–4.0) | 0.0 (0.0–2.0) |
| MDS-UPDRS Part 3 score | 4.0 (1.0–7.0) | 4.0 (1.0–7.0) | 2.0 (0.0–6.0) |
| HVLT-delayed recall score | 46.0 (34.0–56.0) | 48.0 (38.0–56.0) | 51.0 (42.0–58.0) |
| HVLT-recognition discrimination score | 48.0 (42.0–56.0) | 48.0 (44.0–53.0) | 52.0 (43.0–59.0) |
| BJLO score | 12.6 (10.6–14.2) | 12.8 (11.2–14.5) | 12.2 (9.8–13.7) |
| PFT score | 50.0 (43.0–57.0) | 52.0 (43.0–60.0) | 50.0 (44.0–57.0) |
| SFT score | 52.0 (46.0–58.0) | 52.0 (44.8–58.0) | 54.0 (48.0–60.0) |
| TMT-A score | 57.1 (25.2–77.9) | 66.6 (31.9–80.7) | 71.6 (41.7–87.9) |
| TMT-B score | 42.1 (3.5–76.5) | 61.0 (18.7–79.7) | 65.9 (32.6–85.3) |
| SDMT score | 49.2 (43.3–55.8) | 51.0 (46.6–55.9) | 54.0 (47.5–60.0) |
| LNS score | 12.0 (10.0–14.0) | 12.0 (10.0–14.0) | 12.0 (10.0–14.0) |
| BNT score | 12.0 (10.0–14.0) | 12.0 (10.0–14.0) | 12.0 (10.0–14.0) |
| Body mass index, kg/m^2^ | 27.0 (24.4–30.5) | 27.4 (25.0–30.4) | 27.0 (24.0–30.7) |
| NSAID use*, % | 122 (20.9%) | 19 (16.4%) | 114 (20.0%) |
| Total WBC count (×10³/µL) | 5.60 (4.78–6.89) | 5.63 (4.75–6.96) | 2.16 (1.71–2.87) |
| Neutrophil count (×10³/µL) | 3.55 (2.78–4.46) | 3.36 (2.65–4.57) | 3.43 (2.88–4.26) |
| Lymphocyte count (×10³/µL) | 1.51 (1.22–1.85) | 1.52 (1.23–1.84) | 1.56 (1.30–1.91) |
| Neutrophil-lymphocyte ratio | 2.36 91.77–3.05) | 2.22 (1.67–3.07) | 2.16 (1.71–2.87) |

The data are expressed as No. (%) or median value (interquartile range).

*Regular use within 1 month prior to blood sampling.

**Abbreviation:** BJLO = Benton Judgment of Line Orientation; BNT = Boston Naming Test; HVLT = Hopkins Verbal Learning Test; LNS = Letter-Number Sequencing; PFT = Phonemic Fluency Test; MDS-UPDRS = Movement Disorders Society Unified Parkinson’s Disease Rating Scale; PD = Parkinson’s disease; RBD = rem sleep behavior disorder; SDMT = Symbol-Digit Modalities Test; SFT = Semantic Fluency Test; TMT = Trail Making Test; WBC = white blood cell.
