# Supplementary material for: Association of Peripheral Blood Neutrophil‐Lymphocyte Ratio With Motor and Cognitive Function in Prodromal Parkinson's Disease
Source: Brain Behav. 2025 Dec 17;15(12):e71141. doi: 10.1002/brb3.71141 (PMC12712348; doi:10.1002/brb3.71141)
Supplement: Supplementary file 2 — Supplementary Material: brb371141‐Sup‐0002‐TableS2 [file BRB3-15-e71141-s002.docx]

**Supplementary Table 2. Correlates of blood neutrophil and lymphocyte counts and NLR according to distinct prodromal phenotypes**

|  | **Neutrophil** | |  | **Lymphocyte** | |  | **NLR** |  |
| --- | --- | --- | --- | --- | --- | --- | --- | --- |
| **Variables** | **Spearman r (95% CI)** | ***P* value** |  | **Spearman r (95% CI)** | ***P* value** |  | **Spearman r (95% CI)** | ***P* value** |
| **RBD with hyposmia (*n*=583)** | | | | | | | | |
| MDS-UPDRS Part 3 score | 0.008 (–0.077 to 0.094) | 0.854 |  | –0.112 (–0.196 to –0.026) | **0.008** |  | 0.101 (0.026 to 0.194) | **0.015** |
| HVLT-delayed recall score | –0.035 (–0.121 to 0.049) | 0.416 |  | –0.068 (–0.147 to 0.013) | 0.107 |  | 0.015 (–0.072 to 0.095) | 0.728 |
| HVLT-recognition discrimination score | –0.044 (–0.130 to 0.041) | 0.296 |  | –0.011 (–0.102 to 0.072) | 0.787 |  | –0.031 (–0.114 to 0.048) | 0.463 |
| BJLO score | –0.024 (–0.112 to 0.052) | 0.571 |  | –0.040 (–0.120 to 0.042) | 0.352 |  | 0.024 (–0.061 to 0.104) | 0.580 |
| PFT score | –0.035 (–0.120 to 0.051) | 0.410 |  | 0.020 (–0.066 to 0.101) | 0.645 |  | –0.050 (–0.134 to 0.032) | 0.237 |
| SFT score | –0.030 (–0.112 to 0.052) | 0.485 |  | 0.032 (–0.054 to 0.109) | 0.453 |  | –0.051 (–0.134 to 0.041) | 0.231 |
| TMT-A score | 0.015 (–0.069 to 0.105) | 0.720 |  | 0.014 (–0.067 to 0.089) | 0.747 |  | –0.004 (–0.087 to 0.083) | 0.932 |
| TMT-B score | –0.012 (–0.097 to 0.070) | 0.778 |  | 0.015 (–0.063 to 0.096) | 0.721 |  | –0.027 (–0.111 to 0.051) | 0.525 |
| SDMT score | –0.047 (–0.134 to 0.038) | 0.273 |  | –0.004 (–0.088 to 0.083) | 0.934 |  | –0.029 (–0.109 to 0.061) | 0.492 |
| LNS score | –0.017 (–0.100 to 0.065) | 0.683 |  | –0.067 (–0.149 to 0.017) | 0.116 |  | 0.034 (–0.055 to 0.114) | 0.421 |
| BNT score | –0.034 (–0.122 to 0.047) | 0.419 |  | –0.081 (–0.167 to 0.001) | 0.057 |  | 0.045 (–0.041 to 0.128) | 0.292 |
| **RBD only (*n*=116)** | | | | | | | | |
| MDS-UPDRS Part 3 score | 0.070 (–0.128 to 0.273) | 0.473 |  | –0.069 (–0.253 to 0.122) | 0.477 |  | 0.170 (–0.002 to 0.334) | 0.073 |
| HVLT-delayed recall score | –0.086 (–0.267 to 0.118) | 0.376 |  | 0.065 (–0.128 to 0.282) | 0.507 |  | –0.131 (–0.311 to 0.064) | 0.177 |
| HVLT-recognition discrimination score | –0.207 (–0.372 to 0.007) | **0.031** |  | 0.070 (–0.109 to 0.270) | 0.473 |  | –0.237 (–0.424 to –0.028) | **0.013** |
| BJLO score | –0.051 (–0.244 to 0.134) | 0.599 |  | –0.101 (–0.297 to 0.114) | 0.298 |  | 0.038 (–0.167 to 0.252) | 0.700 |
| PFT score | –0.082 (–0.279 to 0.109) | 0.401 |  | –0.209 (–0.385 to –0.030) | **0.030** |  | 0.043 (–0.139 to 0.240) | 0.657 |
| SFT score | –0.163 (–0.337 to 0.023) | 0.093 |  | –0.038 (–0.231 to 0.160) | 0.694 |  | –0.122 (–0.302 to 0.051) | 0.209 |
| TMT-A score | 0.060 (–0.137 to 0.236) | 0.539 |  | –0.113 (–0.314 to 0.091) | 0.246 |  | 0.124 (–0.044 to 0.297) | 0.202 |
| TMT-B score | –0.192 (–0.382 to –0.007) | **0.046** |  | –0.034 (–0.225 to 0.177) | 0.726 |  | –0.128 (–0.307 to 0.051) | 0.185 |
| SDMT score | –0.156 (–0.321 to 0.037) | 0.106 |  | –0.101 (–0.299 to 0.088) | 0.300 |  | –0.089 (–0.270 to 0.085) | 0.358 |
| LNS score | –0.073 (–0.262 to 0.124) | 0.450 |  | –0.146 (–0.328 to 0.039) | 0.133 |  | 0.032 (–0.165 to 0.218) | 0.744 |
| BNT score | 0.001 (–0.177 to 0.180) | 0.993 |  | –0.210 (–0.394 to –0.037) | **0.030** |  | 0.157 (–0.025 to 0.340) | 0.104 |
| **Hyposmia only (*n*=570)** | | | | | | | | |
| MDS-UPDRS Part 3 score | 0.033 (–0.053 to 0.116) | 0.449 |  | –0.068 (–0.155 to 0.015) | 0.115 |  | 0.109 (0.021 to 0.191) | **0.011** |
| HVLT-delayed recall score | –0.022 (–0.108 to 0.074) | 0.614 |  | 0.092 (0.005 to 0.181) | **0.033** |  | –0.104 (–0.194 to –0.010) | **0.016** |
| HVLT-recognition discrimination score | –0.036 (–0.123 to 0.052) | 0.397 |  | 0.095 (0.003 0.175) | **0.028** |  | –0.100 (–0.186 to –0.010) | **0.020** |
| BJLO score | –0.070 (–0.156 to 0.018) | 0.105 |  | –0.041 (–0.129 to 0.040) | 0.346 |  | –0.026 (–0.106 to 0.061) | 0.545 |
| PFT score | 0.074 (–0.007 to 0.162) | 0.083 |  | –0.020 (–0.099 to 0.070) | 0.650 |  | 0.073 (–0.012 to 0.159) | 0.089 |
| SFT score | 0.047 (–0.041 to 0.132) | 0.275 |  | 0.063 (–0.023 to 0.146) | 0.141 |  | –0.025 (–0.112 to 0.054) | 0.556 |
| TMT-A score | –0.082 (–0.170 to –0.001) | 0.057 |  | 0.019 (–0.066 to 0.106) | 0.660 |  | –0.103 (–0.183 to –0.024) | **0.017** |
| TMT-B score | –0.048 (–0.124 to 0.037) | 0.263 |  | –0.029 (–0.116 to 0.055) | 0.504 |  | –0.030 (–0.106 to 0.052) | 0.490 |
| SDMT score | –0.100 (–0.178 to –0.013) | **0.020** |  | 0.015 (–0.064 to 0.099) | 0.731 |  | –0.095 (–0.172 to -0.011) | **0.027** |
| LNS score | –0.022 (–0.108 to 0.061) | 0.612 |  | –0.023 (–0.103 to 0.061) | 0.593 |  | 0.002 (–0.083 to 0.095) | 0.972 |
| BNT score | –0.031 (–0.119 to 0.054) | 0.466 |  | 0.044 (–0.041 to 0.136) | 0.308 |  | –0.066 (–0.151 to 0.013) | 0.126 |

Bold text indicates a statistically significant difference.

**Abbreviation:** BJLO = Benton Judgment of Line Orientation; BNT = Boston Naming Test; CI = confidence interval; HVLT = Hopkins Verbal Learning Test; LNS = Letter-Number Sequencing; PFT = Phonemic Fluency Test; MDS-UPDRS = Movement Disorders Society Unified Parkinson’s Disease Rating Scale; NLR = neutrophil-lymphocyte ratio; PD = Parkinson’s disease; RBD = rem sleep behavior disorder; SDMT = Symbol-Digit Modalities Test; SFT = Semantic Fluency Test; TMT = Trail Making Test.
